# Low-dose retinoic acid enhances in vitro invasiveness of human oral squamous-cell-carcinoma cell lines

**DOI:** 10.1054/bjoc.2001.1862

**Published:** 2001-07

**Authors:** D Uchida, H Kawamata, K Nakashiro, F Omotehara, S Hino, M O Hoque, N-M Begum, H Yoshida, M Sato, T Fujimori

**Affiliations:** Second Department of Oral and Maxillofacial Surgery, Tokushima University School of Dentistry, 3-18-15 Kuramoto, Tokushima, 770-8504, Japan; Department of Surgical and Molecular Pathology, Dokkyo University School of Medicine, 880 Kita-kobayashi, Mibu, Shimo-tsuga, Tochigi, 321-0293, Japan

**Keywords:** oral squamous cell carcinoma, retinoic acid, invasion, tPA, MMP

## Abstract

Retinoids inhibit the proliferation of several types of tumour cells, and are used for patients with several malignant tumours. In this study, we examined the effect of retinoic acids (RAs) on the invasive potentials of the oral squamous cell carcinoma (SCC) cells, BHY and HNt. BHY cells expressed all of retinoid nuclear receptors (RARα, β, γ, and RXRα) and cytoplasmic retinoic acid binding proteins (CRABP1 and CRABP2). HNt cells lacked the expression of RARβ, but expressed other nuclear receptors and CRABPs. All-trans retinoic acid (ATRA) and 13-cis retinoic acid (13-cisRA) (10^−6^and 10^−7^M) inhibited the growth of the cells, but low-dose ATRA and 13-cisRA (10^−8^M) marginally affected the growth of the cells. Surprisingly, low-dose RAs enhanced the activity of tissue-type plasminogen activator (tPA), and activated pro-matrix metalloproteinases (proMMP2 and proMMP9). Activation of proMMP2 and proMMP9 was inhibited by aprotinin, a serine-proteinase, tPA inhibitor. Furthermore, low-dose RAs enhanced the in vitro invasiveness of BHY cells. These results indicate that low-dose RAs enhances the in vitro invasiveness of oral SCC cells via an activation of proMMP2 and proMMP9 probably mediated by the induction of tPA. © 2001 Cancer Research Campaign http://www.bjcancer.com


*British Journal of Cancer* (2001) **85**(1), 122–128
© 2001 Cancer Research Campaign
doi: 10.1054/ bjoc.2001.1862, available online at http://www.idealibrary.com on IDEAL®

### Low-dose retinoic acid enhances in vitro invasiveness of human oral squamous-cell-carcinoma cell lines

**D Uchida[1], H Kawamata[2], K Nakashiro[1], F Omotehara[1], S Hino[1], MO Hoque[1], N-M Begum[1], H Yoshida[1], M Sato[1] and T Fujimori[2]**

[1]Second Department of Oral and Maxillofacial Surgery, Tokushima University School of Dentistry, 3-18-15 Kuramoto, Tokushima 770-8504, Japan; [2]Department of Surgical and Molecular Pathology, Dokkyo University School of Medicine, 880 Kita-kobayashi, Mibu, Shimo-tsuga, Tochigi, 321-0293, Japan

**Summary** Retinoids inhibit the proliferation of several types of tumour cells, and are used for patients with several malignant tumours. In this study, we examined the effect of retinoic acids (RAs) on the invasive potentials of the oral squamous cell carcinoma (SCC) cells, BHY and HNt. BHY cells expressed all of retinoid nuclear receptors (RARα, β, γ, and RXRα) and cytoplasmic retinoic acid binding proteins (CRABP1 and CRABP2). HNt cells lacked the expression of RARβ, but expressed other nuclear receptors and CRABPs. All-trans retinoic acid (ATRA) and 13-cis retinoic acid (13-cisRA) ($10^{-6}$ and $10^{-7}$ M) inhibited the growth of the cells, but low-dose ATRA and 13-cisRA ($10^{-8}$ M) marginally affected the growth of the cells. Surprisingly, low-dose RAs enhanced the activity of tissue-type plasminogen activator (tPA), and activated pro-matrix metalloproteinases (proMMP2 and proMMP9). Activation of proMMP2 and proMMP9 was inhibited by aprotinin, a serine-proteinase, tPA inhibitor. Furthermore, low-dose RAs enhanced the in vitro invasiveness of BHY cells. These results indicate that low-dose RAs enhances the in vitro invasiveness of oral SCC cells via an activation of proMMP2 and proMMP9 probably mediated by the induction of tPA. © 2001 Cancer Research Campaign http://www.bjcancer.com

**Keywords**: oral squamous cell carcinoma; retinoic acid; invasion; tPA; MMP

Retinoids, natural and synthetic vitamin A metabolites and analogues, inhibit the proliferation of several types of tumour cells, such as head and neck squamous cell carcinoma (SCC) (Jetten et al, 1990; Lotan, 1994; Oridate et al, 1996), oesophageal cancer (Xu et al, 1999), lung cancer (Zou et al, 1998; Sun et al, 1999a,b), prostate cancer (Sun et al, 1999c), and cervical cancer (Lotan et al, 1995; Oridate et al, 1997). The effects of retinoids are mediated by both nuclear retinoid receptors (RARs and RXRs) and cytoplasmic retinoic acid binding proteins (CRABP1 and CRABP2) (Pemrick et al, 1994). Retinoids are used as a chemo-preventive or chemotherapeutic agent for patients with several solid tumours, and some successful results from the clinical trials has been reported (Hong et al, 1990; Lippman et al, 1993; Mitchell et al, 1995). Hong et al (1990) reported that 13-cis retinoic acid (13-cisRA) suppressed the oral pre-malignant lesions and decreased the incidence of second primary tumours in head and neck cancer. Furthermore, all-trans retinoic acid (ATRA) is currently used as a standard drug for acute premyelocytic leukaemia, and these successful treatments are recognized as the first example of differentiation therapy (Lo-coco et al, 1998). Molecular mechanisms of the differentiation-inducing action of ATRA on acute premyelocytic leukaemia is well understood (He et al, 1999). On the solid tumours, however, the mechanisms of the action of retinoids was not fully understood.

Oral SCC are characterized by a high degree of local invasion and a high rate of metastasis to cervical lymph nodes, but a low rate of metastases to distant organs. Oral SCC frequently show the local recurrence after initial treatment, probably due to micro-invasion and/or micro-metastasis of the tumour cells at primary site. We recently reported that oral SCC cells produced a large amount of matrix-degrading enzymes and that the net activity of MMP2 (active-MMP2/TIMP2) produced by cancer cells contributed to lymph-node metastasis in a nude mouse orthotopic inoculation model (Kawamata et al, 1997). Furthermore, we demonstrated that active-MMP2 in cancer cell nests played an important role on lymph node metastasis of oral SCC patients by microdissection-zymography of human oral SCC tissues (Kawamata et al, 1998). Thus, MMPs may play an important role on the invasion and metastasis of oral cancer cells.

In order to use retinoids more effectively on the patients with several malignancies, including oral SCC, the effect of retinoids not only on the proliferation of the cells but also on the other biological characteristics, such as invasiveness or angiogenesis, should be examined. In this study, we investigated the effect of retinoids on the invasive potentials of oral SCC cells in vitro. First, we examined the expression of nuclear receptors and cytoplasmic binding proteins for retinoids in oral SCC cell lines, BHY and HNt which were established and characterized in our laboratory (Kawamata et al, 1997). Then we examined the effect of low-dose ATRA ($10^{-8}$ M) and 13-cisRA ($10^{-8}$ M), which did not affect the cell growth, on the expression or activity of the matrix-

*Received 1 December 2000*
*Revised 23 February 2001*
*Accepted 28 March 2001*

*Correspondence to:* H Kawamata

Dr Uchida and Dr Kawamata contributed equally to this paper degrading enzymes, and the in vitro invasiveness of the oral SCC cells.

### MATERIALS AND METHODS

### Cells and cell culture

BHY and HNt (Kawamata et al, 1997) cells were derived from human oral SCC tumours from patients. HT1080 cells are a human fibrosarcoma cell line purchased from Dai-Nippon Seiyaku, Osaka, Japan. All of the cells were maintained in DMEM (Life Technologies, Inc, Gaithersburg, MD) supplemented with 10% FCS (Bio-Whittaker, Walkersville, MD), 100 μg $ml^{-1}$ streptomycin, and 100 U $ml^{-1}$ penicillin, 0.25 μg $ml^{-1}$ Amphotericin B (Life Technologies, Inc.) in a humidified atmosphere of 95% air and 5% $CO_2$ at 37˚C.

### MTT assay

We examined the effect of ATRA (Sigma, St. Louis, MO) and 13-cisRA (Sigma) on the growth of the cells by an assay in which MTT (Sigma) was used. Cells were seeded on a 96-well plate (Falcon; Becton Dickinson Labware, Lincoln Park, NJ) at $5 \times 10^3$ cells per well in DMEM containing 10% FCS. After 24 h, cells were washed twice with DMEM without FCS and cultured in DMEM without FCS in the presence or absence of ATRA ($10^{-6}$, $10^{-7}$ and $10^{-8}$ M) or 13-cisRA ($10^{-6}$, $10^{-7}$ and $10^{-8}$ M). After 3 days, the number of cells was quantitated by MTT assay (Carmichael et al, 1987).

### Gelatinase zymography and reverse gelatinase zymography

Cells were grown to confluence in 60 mm dishes (Falcon) in the serum-supplemented medium, washed twice with serum-free DMEM, and cultured in 3 ml of serum-free DMEM in the presence or absence of ATRA ($10^{-8}$ M) or 13-cisRA ($10^{-8}$ M) for an additional 48 h. The medium was collected, clarified by centrifugation at 1800 ***g***, and concentrated down to 0.1 ml approximately 30-fold by Centricon 10 (cut-off, Mr 10 000; Millipore Corp, Bedford, MA). We have already confirmed that the process of the protein concentration did not activate the progelatinases in the samples (Kawamata et al, 1997). The protein concentration of the samples are determined by Bio-Rad protein assay (Bio-Rad, Hercules, CA). The gels for zymorgraphy were composed of gelatin (0.1%; Sigma) and polyacrylamide (10%), and for reverse zymography, of gelatin (0.1%), polyacrylamide (12.5%) and concentrated conditioned medium (100 μg $ml^{-1}$) of the human fibrosarcoma cell line (HT1080) as a source of gelatinases. 5 μg protein samples from BHY and HNt, and 1 μg protein sample from HT1080, or 10 μg protein samples from all cells for reverse zymography were added to a loading buffer (50 mM Tris-HCl (pH 6.8), 2% SDS, 0.1% bromophenol blue, and 10% glycerol) and were not heated before electrophoresis. Gels were run at 30 mA/gel at 4˚C, incubated overnight at 37˚C in 0.05 M Tris-HCl buffer (pH 7.5) containing 10 mM $CaCl_2$, stained with Coomassie blue (0.25%), and destained in methanol:acetic acid:water (50: 10:40). Clear zones indicated the presence of gelatinolytic activity in zymography, and dark zones indicated the presence of gelatinase inhibitors in reverse zymography.

### RT-PCR

Cells grown in monolayers were harvested at early confluence. RNA was prepared by lysing of cells in a hypotonic buffer containing Nonidet P-40 (Sigma), followed by removal of nuclei. Cytoplasmic RNA was reverse-transcribed by Moloney murine leukaemia virus reverse transcriptase (Life Technologies, Inc) at 42˚C for 60 min using random primer (5 μM; Life Technologies, Inc.) in 20 μl of the reaction mixture. Subsequently, 1 μl of the products was subjected to PCR amplification. PCR was performed as follows: the final concentration of dNTPs and primers in the reaction mixture were 200 μM and 1 μM, respectively. Taq DNA polymerase (Takara Biomedicals, Kusatsu, Japan) was added to the mixture at a final concentration of 0.05 U $\mu l^{-1}$, and the reaction was carried out in a Takara Thermal Cycler MP (Takara Biomedicals) under the following conditions: 94˚C for 3 min and then 94˚C for 1 min, 60˚C for 1.5 min, 72˚C for 2.5 min for 30 cycles, and extension at 72˚C for 4 min. To determine whether conditions were adequate for semi-quantitative RT-PCR, we reverse-transcribed 0.05, 0.5, 5 and 10 μg of total RNA and amplified the fragments under the same PCR conditions with different numbers of cycles (20, 24, 28, 32 and 36 cycles). We used 5 μg of cytoplasmic RNA as a template for reverse transcription and the indicated cycles on Table 1 for each genes. The primers used were also listed in Table 1.

### Chromogenic assay for tPA activity

Chromogenic assay for tPA activity was performed by Spectrolyse/fibrin kit (Biopool, Ventura, CA) according to the manufacturer's instructions. Conditioned medium of all cells was collected and prepared as described above. Membrane extracts were prepared as follows: cells were lysed in 20 mM HEPES-HCl (pH 7.5), 150 mM NaCl, 2 mM $CaCl_2$, 1 mM phenyl-methylsulfonyl fluoride, and 2 μg $ml^{-1}$ of leupeptin. After centrifugation of the lysates at 700 ***g*** for 20 min at 4˚C, the supernatant was further ultracentrifuged at 100 000 ***g*** for 1 h at 4˚C. The pellet was resuspended in 50 mM Tris-HCl (pH 7.5) containing 0.5% Triton X-100. The protein concentration of samples were determined by

**Table 1** Primers used

| | Upper-stream | Down-stream | Length of DNA frragment (bp) | Cycles |
|---|---|---|---|---|
| RAR-α | gtctttgccttcgccaaccag | gccctctgagttctccaaca | 333 | 26 |
| RAR-β | ggagacttcgaagcaagaatg | aacacaaggtcagtcagaggac | 452 | 28 |
| RAR-γ | tgacccagtatgtagaagccag | gttccggtcatttcgcacagc | 678 | 28 |
| RXR-α | atcagcaaagacctcagccgc | aggctctgggtgaacaacgctg | 744 | 35 |
| CRABP1 | ctacatcaagacatccaccaccg | ctagggatacaagaggcaccaag | 446 | 33 |
| CRABP2 | ggtgaatgtgatgctgaggaag | ctacagggacaaagggtagaag | 706 | 33 |

© 2001 Cancer Research Campaign

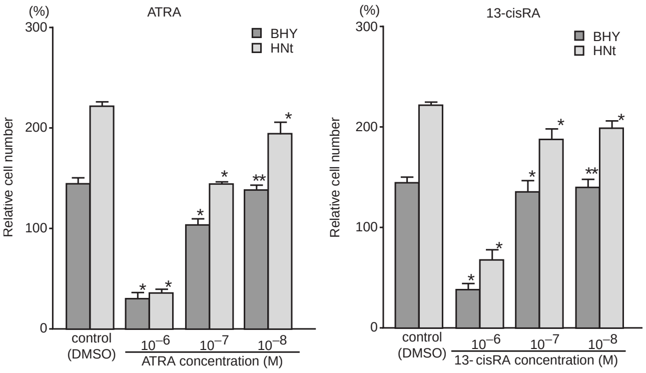

**Figure 1** Effect of RAs on in vitro growth of oral SCC cells. The columns show the average of 8 samples and the bars show SD. Data are representative of 2 separate experiments with similar results. *, $P < 0.01$; **, not significant when compared to that of control by one-way ANOVA

Bio-Rad protein assay (Bio-Rad). 500 ng protein samples from conditioned medium and plasma membrane were subjected to the chromogenic assay.

### In vitro invasion assay

Oral SCC cells, $2 \times 10^5$ cells in Experiment I or $5 \times 10^4$ cells in Experiment II, were seeded on Matrigel-coated polycarbonate filters of 8 µm pore size in triplicate Transwell invasion chambers (6.5 mm in diameter) (Falcon). Then, ATRA or 13-cisRA at $10^{-8}$ M was added in the upper compartment. After 48 h incubation, the cells and Matrigel on the upper-surface of the membrane were wiped out with a cotton swab, and the membrane was removed from the chamber, and stained with haematoxylin-eosin. Plugged cells in the pore or the cells attached on the lower-surface of the membrane were counted in 10 fields at high power view (× 400) in Experiment I, or in total area of the membrane in Experiment II by a third person without any knowledge of the treatments.

### RESULTS

### Effect of ATRA and 13-cisRA on the growth of oral SCC cells in vitro

Both ATRA and 13-cisRA inhibited the growth of BHY and HNt cells in a dose-dependent manner (Figure 1). The cell number of BHY at day 3 was decreased down to 20% and 30% of the control level (the cell number at day 0) by ATRA ($10^{-6}$ M) and 13-cisRA ($10^{-6}$ M), respectively. On the other hand, the cell number of HNt was decreased down to 30% and 60% of the control level by ATRA ($10^{-6}$ M) and 13-cisRA ($10^{-6}$ M), respectively. However, low-dose ATRA ($10^{-8}$ M) and 13-cisRA ($10^{-8}$ M) did not affect the growth of BHY cells at all, and minimally affected the growth of HNt cells (Figure 1). Data are representative of 2 separate experiments with similar results.

### Expression of retinoid nuclear receptors and cytoplasmic retinoic acid binding proteins in oral SCC cells

We examined the expressions of mRNA for retinoid nuclear receptors and cytoplasmic retinoic acid binding proteins in oral SCC

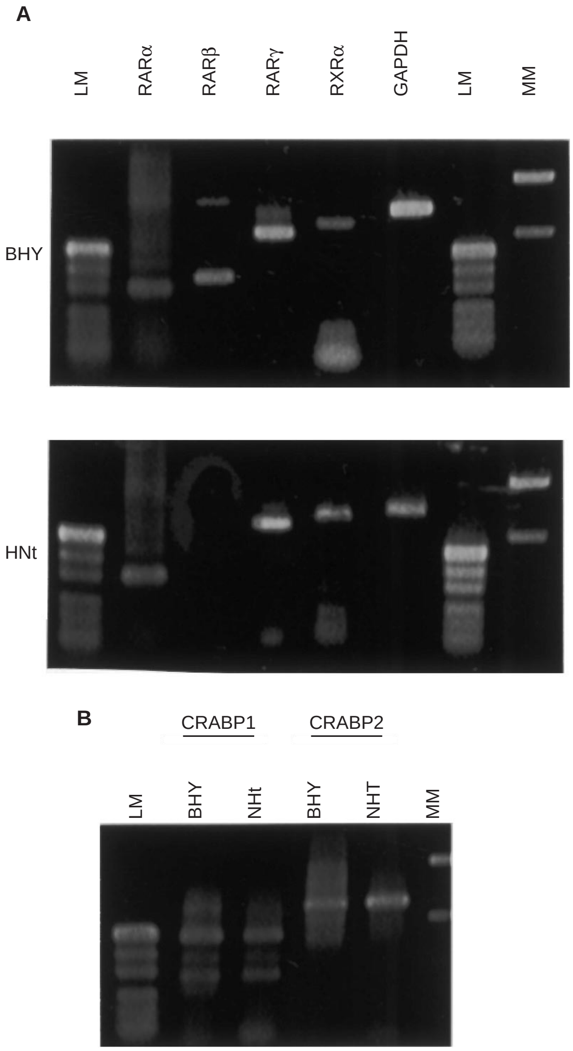

**Figure 2** Expression of mRNA for retinoid nuclear receptors (panel **A**) and cytoplasmic retinoic acid binding proteins (panel **B**) in oral SCC cells determined by semi-quantitative RT-PCR. *GAPDH* was used as an internal control. LM; low molecular-weight marker, Hap2-digested pUC19 (501, 489, 404, 331, 242, 190, 147, 111, 110 bp), MM; Pvu2-digested pBK-CMV plasmid (1026, 608 bp)

cells by semi-quantitative RT-PCR method. BHY cells expressed all of RARs (RARα, β and γ), RXRα, (Figure 2A) and CRABP1 and CRABP2 (Figure 2B). On the other hand, HNt cells lacked the expression of RARβ, but expressed RARα, RARγ, RXRα, (Figure 2A) and CRABP1 and CRABP2 (Figure 2B). We sequenced all of the fragments, and confirmed the fragments were amplified from the genes of our interest. (data not shown).

### Activation of proMMPs by treatment with low-dose ATRA and 13-cisRA in oral SCC cells

We reported that BHY and HNt cells expressed several types of MMP at very high level (Kawamata et al, 1997). Then, we examined the effect of low-dose RAs on the expression and the activation of MMPs. When we treated BHY and HNt cells by ATRA ($10^{-8}$ M) or 13-cisRA ($10^{-8}$ M) for 48 h, the gelatinolytic band of proMMP9 moved slightly faster than that in the untreated controls,

 © 2001 Cancer Research Campaign

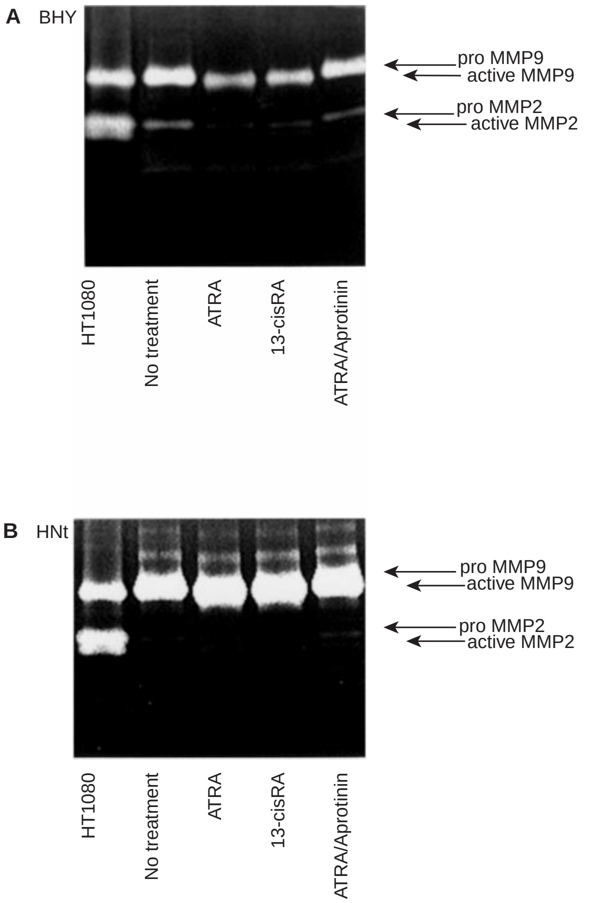

**Figure 3** Gelatin zymography, **A**; BHY, **B**; HNt. The gels were composed of gelatin (0.1%) and polyacrylamide (10%). Aliquots (5 μg protein from BHY and HNt, 1 μg from HT1080) of conditioned medium were electrophoresed. The gels were incubated overnight at 37˚C in 0.05 M Tris-HCl buffer (pH 7.5) containing 10 mM $CaCl_2$, stained with Coomassie blue (0.25%), and destained. Clear zones indicated the presence of gelatinolytic activity

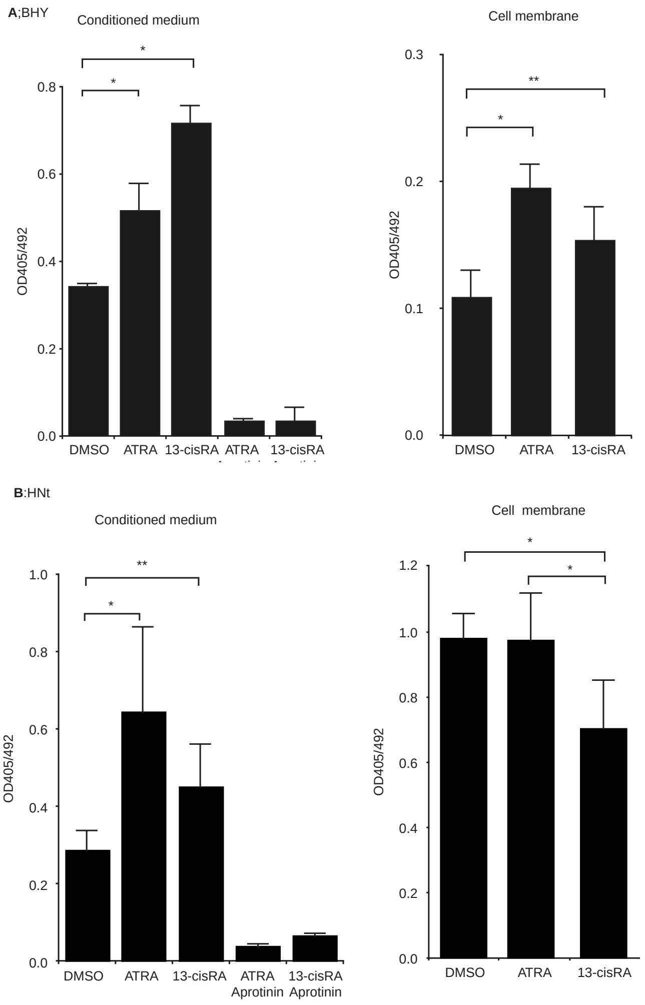

**Figure 4** Chromogenic assay for tPA activity (panels **A** and **B**). The columns show the average of 3 samples and the bars show SD. Data are representative of 2 separate experiments with similar results. *, $P < 0.05$; **, not significant (one-way ANOVA)

and the movement of the bands in the treated cells corresponded to that in HT1080 cells (Figure 3A, B). Then, the shifted bands were considered as the proteolytically activated form of MMP9. Furthermore, the intensity of proMMP2 bands in BHY and HNt cells was decreased, and the proteolytically activated form of MMP2 apparently appeared by treatment with ATRA ($10^{-8}$ M) or 13-cisRA ($10^{-8}$ M) (Figure 3A, B). The activation of proMMPs by RAs in BHY and HNt cells was inhibited by treatment with a serine proteinase inhibitor, aprotinin (Figure 3A, B). Data are representative of 3 separate experiments with similar results.

### Effect of RAs on the activity of tPA and uPA in oral SCC cells

We examined the effect of low-dose RAs ($10^{-8}$ M) on tPA activity in the oral SCC cells. ATRA significantly ($P < 0.05$; one-way ANOVA) increased the activity of tPA in the medium as well as on the plasma membrane of BHY and HNt cells (Figure 4A, B). 13-cisRA also significantly ($P < 0.05$; one-way ANOVA) increased the activity of tPA in the medium of BHY cells and on the plasma membrane of HNt cells (Figure 4A, B). 13-cisRA slightly increased the activity of tPA on the plasma membrane of BHY and in the medium of HNt, although the effect of 13-cisRA on these cells did not reach the significant level (Figure 4A, B). Data are representative of 2 separate experiments with similar results. We also examined the effect of RAs on the activity of uPA in the medium of the oral SCC cells by uPA zymography. However, low-dose RAs did not affect the activity of uPA from oral SCC cells (data not shown).

### Effect of RAs on the expression of MT1-MMP and the activity of TIMPs in oral SCC cells

We previously reported that BHY and HNt cells expressed MT1-MMP and TIMPs (TIMP1 and TIMP2) (Kawamata et al, 1997). Then, we examined the effect of low-dose RAs ($10^{-8}$ M) on the expression of MT1-MMP and the activity of TIMPs in the oral SCC cells. The expression of MT1-MMP was examined by semi-quantitative RT-PCR, and the activity of TIMPs was examined by reverse-zymography. The expression of MT1-MMP and the activity of TIMPs (TIMP1 and TIMP2) were not affected by low-dose RAs (data not shown).

© 2001 Cancer Research Campaign 

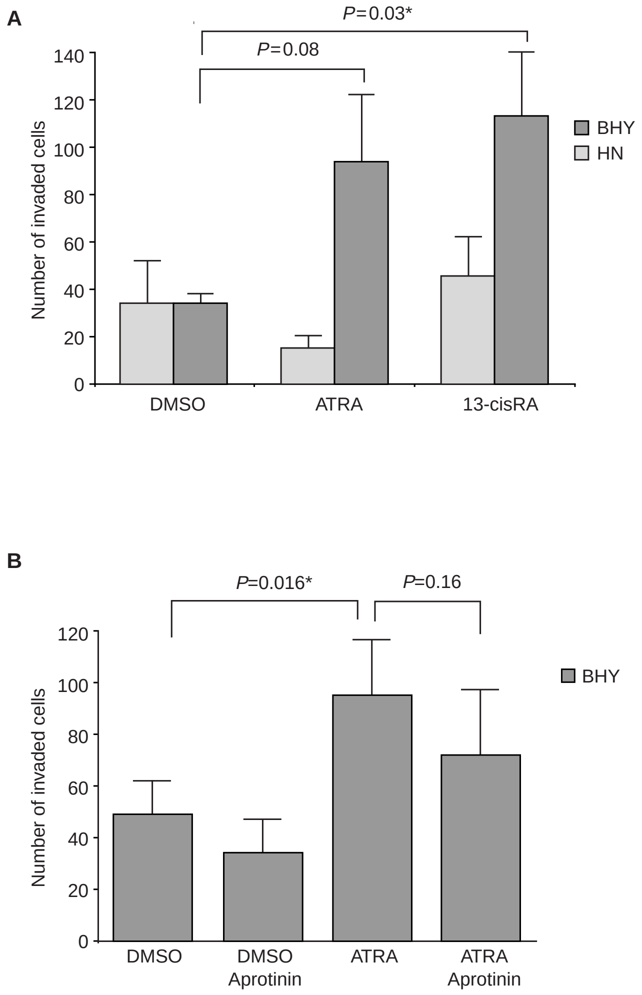

**Figure 5** In vitro invasion assay. Panel **A**, experiment I; panel **B**, experiment II. The columns show the average of 3 samples and the bars show SD. Data are representative of 2 separate experiments with similar results. *, statistically significant (one-way ANOVA)

### Effect of RAs on the invasion of oral SCC cells

We examined the effect of low-dose RAs ($10^{-8}$ M) on in vitro invasiveness of the oral SCC cells. We repeated the Experiment I twice, and Experiment II 4 times, and obtained similar results from these separate experiments. Here, we presented the representative results from the Experiment I and Experiment II. In Experiment I, 13-cisRA significantly enhanced the in vitro invasiveness of BHY cells ($P$ = 0.03; one-way ANOVA) (Figure 5A). ATRA also enhanced the invasiveness of BHY cells, but the effect of ATRA did not reach the significant level ($P$ = 0.08; one-way ANOVA) (Figure 5A). Both ATRA and 13-cisRA did not affect the invasiveness of HNt cells (Figure 5A). In Experiment II, we further examined the effect of ATRA on the invasiveness of BHY cells. In this experimental condition, ATRA significantly enhanced the invasiveness of BHY cells ($P$ = 0.016; one-way ANOVA) (Figure 5B). A serine proteinase inhibitor, aprotinin slightly inhibited the effect of ATRA, although the effect did not reach the significant level ($P$ = 0.16; one-way ANOVA) (Figure 5B). If we increased the concentration of aprotinin, aprotinin affects the viability of the cells. Then, we could not demonstrate the apparent inhibitory effect of aprotinin on the action of RAs.

### DISCUSSION

In this investigation, we examined the effect of retinoic acids on the growth and the invasive potentials of the oral SCC cells in vitro. We demonstrated that ATRA ($10^{-6}$ and $10^{-7}$ M) and 13-cisRA ($10^{-6}$ and $10^{-7}$ M) inhibited the growth of the oral SCC cells (BHY and HNt) in vitro, but low-dose ATRA ($10^{-8}$ M) and 13-cisRA ($10^{-8}$ M) marginally affect the growth of the cells. Low-dose RAs ($10^{-8}$ M) enhanced the activity of tPA, and converted proMMP2 and proMMP9 to active forms. Furthermore, low-dose RAs enhanced the in vitro invasiveness of BHY cells.

Several investigators reported that retinoids inhibited the invasion of tumour cells. Hendrix et al (1990) and Wood et al (1990) noted that retinoic acid inhibited the invasiveness of melanoma cells by the down-regulation of gelatinases, tPA, and a motility factor receptor, and up-regulation of laminin receptor. Yamamoto et al (1995) reported that retinoic acid inhibited the expression of MMP7 in colon cancer cells. Webber and Waghray (1995) also demonstrated that retinoic acid inhibited the invasion of prostate cancer by the inhibition of uPA activity. Furthermore, Vermenler et al (1995) reported that retinoids stimulated the cell to cell adhesion of cancer cells, and inhibited the invasion of these cancer cells in vitro. However, retinoids were used at the concentrations which might affect the cell growth as well as the invasion in the most of the experiments reported. In this study, we used the RAs at very low concentration ($10^{-8}$ M), which did not affect the cell growth, to see the effect of RAs on the invasiveness of the cells. Before we started the experiment, we expected that low-dose RAs may inhibit the expression and activity of MMPs and in vitro invasiveness of the cells. However, contrary to our expectation, low-dose RAs apparently enhanced the in vitro invasiveness of the cells. These observations were not limited to our oral SCC cells. Tiberio et al (1997) recently reported that ATRA enhanced the invasion of neuroblastoma cells by the induction of tPA.

It is well known that RAs at the physiological concentration enhance the activity of uPA and tPA (Bulen et al, 1997; Lansink et al, 1998). RA-activated retinoic acid receptors directly bind to the retinoic acid responsive element on the tPA gene promoter, and induce the transcriptional activation of tPA gene (Bulen et al, 1997). Plasminogen activators (uPA and tPA), and plasmin, which was proteolytically produced by the plasminogen activators, can activate the latent TGF-β and its family members (Imai et al, 1997), HGF (Mars et al, 1993), and proMMPs (Baramova et al, 1997). Thus, RAs enhance the migration, invasion and differentiation of the cells, and may act as a morphogen during the embryogenesis and the development. Therefore, our observations were not conflicting with the previous reports concerning the function of RAs.

Lotan and his colleagues reported that several cancer cells lacked the expression of RARβ, and the responsiveness of the cancer cells to retinoids were highly dependent on the expression of RARβ (Lotan, 1997; Wan et al, 1999). Then they concluded that RARβ may be a candidate of the tumour suppressor gene (Lotan, 1997). In our oral SCC cells, BHY cells expressed all of RARs (RARα, β, and γ), RXRα, and CRABPs (CRABP1 and CRABP2). HNt cells, however, lacked the expression of RARβ, but expressed other nuclear receptors and CRABPs. BHY cells were derived from highly differentiated SCC, and HNt cells were derived from poorly differentiated SCC (Kawamata et al, 1997). BHY cells formed highly differentiated squamous cell carcinoma, but did not metastasize to any distant organs in nude mice (Kawamata et al,

*© 2001 Cancer Research Campaign*

1997). HNt tumours in nude mice were poorly differentiated squamous cell carcinoma, and frequently metastasized to cervical lymph nodes (Kawamata et al, 1997). Thus, the lack of RARβ may partially account for the aggressive behaviour of HNt cells in nude mice. Furthermore, the different expression pattern of the RA receptors may contribute the different responsiveness of BHY and HNt cells to RAs on the in vitro invasion assay. Low-dose RAs apparently enhanced the invasiveness of BHY cells, but the effect of low-dose RAs on the invasiveness of HNt cells was not demonstrable in this experimental condition, although low-dose RAs enhanced the activity of tPA and activation of proMMPs in both cells. The other factors, such as TGF-β and HGF may affect the in vitro invasiveness of the cells. Bracke et al (1992) reported that ATRA affected the invasiveness of human mammary carcinoma variants (MCF-7/6 and MCF-7/AZ) from the same parental cells (MCF-7) in opposite directions. They showed that treatment with ATRA ($10^{-6}$ M) inhibited the invasion and ruffling of MCF7/6 cells, while the same treatment enhanced the invasion and ruffling of MCF-7/AZ cells. Their published findings may support our present observations.

Retinoids are now used as a chemopreventive or chemotherapeutic agent for patients with several solid tumours (Hong et al, 1990; Lippman et al, 1993; Mitchell et al, 1995). Furthermore, a triple combination of 5-fluorouracil, vitamin A and cobalt-60 radiation, so-called FAR therapy, has been applied to treat the patients with head and neck tumours (Komiyama et al, 1978). If the intratumoral concentration of RAs does not reach the antiproliferative dose, RAs might enhance the invasion and migration of the cancer cells. Then RAs should be used at enough high concentration to show the anti-proliferative effect or differentiation-inducing effect on the cancer cells.

### ACKNOWLEDGEMENTS

This study was supported in part by a Grant-in aid from the Ministry of Education, Science and Culture of Japan.

### REFERENCES

Baramova EN, Bajou K, Remacle A, L'Hoir C, Krell HW, Weidle UH, Noel A and Foidart JM (1997) Involvement of PA/plasmin system in the processing of pro-MMP-9 and in the second step of pro-MMP-2 activation. *FEBS Lett* **405**: 157–162

Bracke ME, Van Larebeke NA, Vyncke BM and Mareel MM (1991) Retinoic acid modulates both invasion and plasma membrane ruffling of MCF-7 human mammary carcinoma cells in vitro. *Br J Cancer* **63**: 867–872

Bulens F, Merchiers P, Ibanez-Tallon I, De Vriese A, Nelles L, Claessens F, Belayew A and Collen D (1997) Identification of a multihormone responsive enhancer far upstream from the human tissue-type plasminogen activator gene. *J Biol Chem* **272**: 663–671

Carmichael J, DeGraff WG, Gazdar AF, Minna JD and Mitchell JB (1987) Evaluation of a tetrazolium-based semiautomated calorimetric assay: assessment of chemosensitivity testing. *Cancer Res* **47**: 936–942

He LZ, Merghoub T and Pandolfi PP (1999) In vivo analysis of the molecular pathogenesis of acute promyelocytic leukemia in the mouse and its therapeutic implications. *Oncogene* **18**: 5278–5292

Hendrix MJ, Wood WR, Seftor EA, Lotan D, Nakajima M, Misiorowski RL, Seftor RE, Stetler-Stevenson WG, Bevacqua SJ, Liotta LA, Sobel ME, Raz A and Lotan R (1990) Retinoic acid inhibition of human melanoma cell invasion through a reconstituted basement membrane and its relation to decreases in the expression of proteolytic enzymes and motility factor receptor. *Cancer Res* **50**: 4121–4130

Hong WK, Lippman SM, Itri LM, Karp DD, Lee JS, Byers RM, Schantz SP, Kramer AM, Lotan R, Peters LJ, Dimery IW, Bown BW and Goepfert H (1990) Prevention of second primary tumors with isotretinoin in squamous-cell carcinoma of the head and neck. *N Engl J Med* **323**: 795–801

Imai S, Okuno M, Moriwaki H, Muto Y, Murakami K, Shudo K, Suzuki Y and Kojima S (1997) 9,13-di-cis-Retinoic acid induces the production of tPA and activation of latent TGF-beta via RAR alpha in a human liver stellate cell line, LI90. *FEBS Lett* **411**: 102–106

Jetten AM, Kim JS, Sacks PG, Rearick JI, Lotan D, Hong WK and Lotan R. (1990) Inhibition of growth and squamous-cell differentiation markers in cultured human head and neck squamous carcinoma cells by beta-all-trans retinoic acid. *Int J Cancer* **45**: 195–202

Kawamata H, Nakashiro K, Uchida D, Harada K, Yoshida H and Sato M (1997) Possible contribution of active MMP2 to lymph-node metastasis and secreted cathepsin L to bone invasion of newly established human oral-squamous-cancer cell lines. *Int J Cancer* **70**: 120–127

Kawamata H, Uchida D, Hamano H, Kimura-Yanagawa T, Nakashiro K, Hino S, Omotehara F, Yoshida H and Sato M (1998) Active-MMP2 in cancer cell nests of oral cancer patients: correlation with lymph node metastasis. *Int J Oncol* **13**: 699–704

Komiyama S, Hiroto I, Ryu S, Nakashima T, Kuwano M and Endo H (1978) Synergistic combination therapy of 5-fluorouracil, vitamin A and cobalt-60 radiation upon head and neck tumors. *Oncology* **35**: 253–257

Lansink M, Koolwijk P, van Hinsbergh V and Kooistra T (1998) Effect of steroid hormones and retinoids on the formation of capillary-like tubular structures of human microvascular endothelial cells in fibrin matrices is related to urokinase expression. *Blood* **92**: 927–938

Lippman SM, Glisson BS, Kavanagh JJ, Lotan R, Hong WK, Paredes-Espinoza M, Hittelman WN, Holdener EE and Krakoff IH (1993) Retinoic acid and interferon combination studies in human cancer. *Eur J Cancer* **29A**: S9–13

Lo-Coco F, Nervi C, Avvisati G and Mandelli F (1998) Acute promyelocytic leukemia: a curable disease. *Leukemia* **12**: 1866–1880

Lotan R (1994) Suppression of squamous cell carcinoma growth and differentiation by retinoids. *Cancer Res* **54**(Suppl): 1987s–1990s

Lotan R (1997) Retinoids and chemoprevention of aerodigestive tract cancers. *Cancer Metastasis Rev* **16**: 349–356

Lotan R, Dawson MI, Zou CC, Jong L, Lotan D and Zou CP (1995) Enhanced efficacy of combinations of retinoic acid-and retinoid X receptor-selective retinoids and alpha-interferon in inhibition of cervical carcinoma cell proliferation. *Cancer Res* **55**: 232–236

Mars WM, Zarnegar R and Michalopoulos GK (1993) Activation of hepatocyte growth factor by the plasminogen activators uPA and tPA. *Am J Pathol* **143**: 949–958

Mitchell MF, Hittelman WK, Lotan R, Nishioka K, Tortolero-Luna G, Richards-Kortum R, Wharton JT and Hong WK (1995) Chemoprevention trials and surrogate end point biomarkers in the cervix. *Cancer* **76**: 1956–1977

Oridate N, Lotan D, Xu XC, Hong WK and, Lotan R (1996) Differential induction of apoptosis by all-trans-retinoic acid and N-(4-hydroxyphenyl) retinamide in human head and neck squamous cell carcinoma cell lines. *Clin Cancer Res* **2**: 855–863

Oridate N, Suzuki S, Higuchi M, Mitchell MF, Hong WK and Lotan R (1997) Involvement of reactive oxygen species in N-(4-hydroxyphenyl) retinamide-induced apoptosis in cervical carcinoma cells. *J Natl Cancer Inst* **89**: 1191–1198

Pemrick SM, Lucas DA and Grippo JF (1994) The retinoid receptors. *Leukemia* **8**: 1797–1806

Sun SY, Li W, Yue P, Lippman SM, Hong WK and Lotan R (1999a) Mediation of N-(4-hydoxyphenyl) retinamide-induced apoptosis in human cancer cells by different mechanisms. *Cancer Res* **59**: 2493–2498

Sun SY, Yue P, Wu GS, El-Deiry WS, Shroot B, Hong WK and Lotan R (1999b) Mechanisms of apoptosis induced by the synthetic retinoid CD437 in human non-small cell lung carcinoma cells. *Oncogene* **18**: 2357–2365

Sun SY, Yue P and Lotan R (1999c) Induction of apoptosis by N-(4-hydroxyphenyl) retinamide and its association with reactive oxygen species, nuclear retinoic acid receptors, and apoptosis-related genes in human prostate carcinoma cells. *Mol Pharmacol* **55**: 403–410

Tiberio A, Farina AR, Tacconelli A, Cappabianca L, Gulino A and Mackay AR (1997) Retinoic acid-enhanced invasion through reconstituted basement membrane by human SK-N-SH neuroblastoma cells involves membrane-associated tissue-type plasminogen activator. *Int J Cancer* **73**: 740–748

Vermeulen SJ, Bruyneel EA, van Roy FM, Mareel MM and Bracke ME (1995) Activation of the E-cadherin/catenin complex in human MCF-7 breast cancer cells by all-trans-retinoic acid. *Br J Cancer* **72**: 1447–1453

Wan H, Oridate N, Lotan D, Hong WK and Lotan R (1999) Overexpression of retinoic acid receptor beta in head and neck squamous cell carcinoma cells increases their sensitivity to retinoid-induced suppression of squamous differentiation by retinoids. *Cancer Res* **59**: 3518–3526

Webber MM and Waghray A (1995) Urokinase-mediated extracellular matrix degradation by human prostatic carcinoma cells and its inhibition by retinoic acid. *Clin Cancer Res* **1**: 755–761

Wood WR, Seftor EA, Lotan D, Nakajima M, Misiorowski RL, Seftor RE, Lotan R and Hendrix MJ (1990) Retinoic acid inhibits human melanoma tumor cell invasion. *Anticancer Res* **10**: 423–432

Xu XC, Liu X, Tahara E, Lippman SM and Lotan R (1999) Expression and up-regulation of retinoic acid receptor-beta is associated with retinoid sensitivity and colony formation in esophageal cancer cell lines. *Cancer Res* **59**: 2477–2483

Yamamoto H, Itoh F, Hinoda Y and Imai K (1995) Suppression of matrilysin inhibits colon cancer cell invasion in vitro. *Int J Cancer* **61**: 218–222

Zou CP, Kurie JM, Lotan D, Zou CC, Hong WK and Lotan R (1998) Higher potency of N-(4-hydroxyphenyl) retinamide than all-trans-retinoic acid in induction of apoptosis in non-small cell lung cancer cell lines. *Clin Cancer Res* **4**: 1345–1355

 *© 2001 Cancer Research Campaign*
